# Pre-implementation factors for Active Music Engagement (AME): barriers and facilitators to integrating an evidence-based dyadic intervention into pediatric oncology care

**DOI:** 10.1186/s43058-026-00979-y

**Published:** 2026-06-03

**Authors:** Kristin M. Story, Elizabeth Harman, Kristin Stegenga, Jelena Golubovic, Sheri L. Robb

**Affiliations:** 1 Center for Health Information and Communication, Richard L. Roudebush VA Medical Center, Indianapolis, USA; 2 School of Nursing, Indiana University, Indianapolis, USA; 3 Children’s Mercy Hospital, Kansas City, USA; 4 School of Medicine, Indiana University, Indianapolis, IN USA

**Keywords:** Music therapy, Implementation, Pediatrics, Oncology

## Abstract

**Background:**

Pediatric cancer treatment is a difficult and distressing experience that often results in sustained, interrelated distress in young children and parents, and traumatic stress symptoms for some parents. Few evidence-based interventions address this dyadic distress. Active Music Engagement (AME) is a manualized, evidence-based music play intervention that significantly reduces distress in child/parent dyads and mitigates traumatic stress and improves well-being among highly distressed parents and those experiencing sociodemographic risk. Successfully integrating AME into standard care requires a systematic understanding of implementation factors. This study aimed to identify site-specific barriers and facilitators that influenced successful AME delivery during a recently completed randomized controlled trial, leveraging multidisciplinary team insights to inform the selection of implementation strategies needed to successfully integrate AME into standard care.

**Methods:**

Semi-structured interviews (*n* = 10) were conducted with study personnel responsible for coordination and delivery of AME in a previous multisite randomized controlled trial; this included Advanced Practice Nurses and Board-Certified Music Therapists from our three participating sites. Interview questions were informed by the Consolidated Framework for Implementation Research. Data were analyzed using Rapid Qualitative Analysis and reported following the Planning for and Assessing Rigor in Rapid Qualitative Analysis framework.

**Results:**

Findings highlighted that AME was strongly supported by staff with direct exposure to the intervention. Key barriers were primarily structural, including limited clinic space, competing demands in the outpatient setting, and limited awareness of the music therapy role among executive leadership. Key facilitators included the intervention’s strong theoretical structure, the perceived high compatibility of AME principles with inpatient care needs, and the value of peer support/collaboration among music therapists. Participants recommended aligning AME delivery with existing inpatient infrastructure, enhancing referral and prioritization systems to manage demand, and refining therapist training to prioritize core theoretical constructs over manualized content.

**Conclusion:**

Future research and implementation efforts should focus on integrating AME into routine inpatient pediatric care, where service structure and patient/parent needs compatibility are highest. Strategies should include broader engagement with hospital leadership and parents, a clear plan for staffing/resource allocation, modified intervention training that supports flexible delivery, and the development of structured decision-support tools for targeted patient referral.

**Supplementary Information:**

The online version contains supplementary material available at 10.1186/s43058-026-00979-y.

**Table:** 

**Contributions to the literature**
• This paper offers a rare example of a pediatric hematology/oncology study using a staged, pre-implementation approach to advance a behavioral health intervention beyond efficacy trials, helping ensure its eventual uptake and routine use.
• By applying an implementation framework, we provide context-specific data on barriers and facilitators for a dyadic, evidence-based music therapy, filling a gap in the literature on family-centered interventions.
• Findings highlight the importance of leadership engagement, infrastructure alignment, and therapist training to promote sustainability.
• The study provides practical strategies for pre-implementation assessment, guiding others who aim to successfully transition complex interventions into routine hospital care.

## Introduction

Pediatric cancer, even in the best circumstances, is a difficult and distressing experience for children and their families. Traumatic stress symptoms (TSS) are common for children and parents with up to 40% of children and between 40 and 83% of parents reporting TSS after cancer treatment [1–3]. Child and parent distress are highly interrelated and interdependent, particularly with young children [4]. Despite this shared burden, there are few evidence-based interventions that specifically address this interrelated distress at the dyad level [5–7].

The dyadic intervention, Active Music Engagement (AME), was developed to address this critical gap. AME is a manualized, music-based intervention for children (3–8 years old) with cancer and their parents. Delivered by board-certified music therapists (MTs) and based in the Contextual Support Model of Music Therapy [8], AME uses dyadic music play to foster a supportive environment characterized by autonomy, structure, and relationship support.

AME has been developed and evaluated in both inpatient and outpatient settings [8–15]. Results from four studies, including two multi-site randomized controlled trials, have established AME’s efficacy and the subgroup of parent/child dyads who derive the most benefit. Specifically, participation in AME has been associated with significant reductions in child symptom severity (anxiety, fatigue, mood, pain) [10], decreases in parent distress (anxiety, fatigue, mood), and mitigates traumatic stress symptoms while improving well-being in parents who report high baseline distress [12] or who report experiencing greater sociodemographic risk [16, 17].

These significant clinical outcomes provide a strong rationale for incorporating AME into standard music therapy practice within pediatric hematology/oncology settings. However, integrating an evidence-based intervention into practice requires a systematic implementation approach. Pre-implementation studies are therefore essential, as they provide contextual insights into potential barriers and facilitators to adoption and inform the selection of tailored implementation strategies that promote long-term fidelity and sustainability. This work is particularly important given the limited number of pediatric hematology/oncology behavioral health interventions that have successfully transitioned from controlled efficacy trials to large-scale implementation, making this study a meaningful contribution to the field [18, 19].

The most recent AME investigation, the multi-site randomized controlled trial Biologic Mechanisms and Dosing of Active Music Engagement (Bio-Muse) (R01NR019190), provided the context for this pre-implementation assessment. The Bio-Muse trial examined the effects, dosing, and biological mechanisms of AME for young children with acute lymphoblastic leukemia and their parent or caregiver, with dyads receiving 30-minute sessions during weekly outpatient visits [13]. Detailed fidelity procedures, including protocol standardization, therapist training, recurring team calls, and self- and external quality monitoring, are described in [20].

The Bio-Muse research team, comprising music therapists (MTs), advance practice nurses (APNs), and other supportive personnel, coordinated and/or delivered the intervention. This diverse, multi-site team offered a unique opportunity to examine the experience of AME delivery and identify factors influencing success, such as the use of standardized music play experiences and materials, including distribution of music-play kits to support home use. MTs, in particular, can provide valuable insight into which elements of training and the manualized intervention should be retained or adapted for large-scale implementation.

The current study aimed to identify barriers and facilitators that influenced successful AME delivery during the Bio-Muse trial. The perspectives from the multidisciplinary team will be used to inform selection of implementation strategies to support future integration of AME into standard care.

## Methods

We conducted semi-structured interviews with study personnel from the three participating sites who were directly involved in AME scheduling and delivery during the Bio-Muse trial. Interviewed personnel fell into two distinct groups: APNs and MTs. APNs held full-time clinical positions at a participating hospital and were responsible for participant recruitment and coordination of study activities. MTs held full or part-time positions at a participating hospital and were responsible for AME delivery [13].

Interview questions were developed and informed by The Consolidated Framework for Implementation Research (CFIR) [21]. CFIR is a well-established framework used to identify factors that influence intervention implementation and effectiveness. Our questions focused on specific constructs within three CFIR domains (Inner Setting; Individual Roles and Relationships; Implementation Process) to explore factors potentially influencing AME delivery and uptake. See Table 2 for CFIR domain definitions and rationale for their inclusion. See supplementary material for the interview guide.

### Participating sites

Participating sites included three U.S. pediatric hematology/oncology programs located within freestanding children’s hospitals across three major Midwestern cities. All hospitals were members of the Children’s Oncology Group (COG), ensuring delivery of protocol-based pediatric oncology treatment and supportive care that is standardized across institutions. Programs were comparable in size, with a median of 31 outpatient beds (range: 21–47) and 38 inpatient beds (range: 37–48). All sites had well-established inpatient music therapy programs (median duration: 4–6 years). However, the trial referenced in these interviews was conducted in the outpatient setting, where music therapy was not routinely available as a standard care service at any site.

### Data collection

An initial invitation to participate was emailed to eligible personnel. Interviews were then scheduled with those who expressed interest. Each interview lasted approximately 45 min and was conducted virtually using a university-approved, HIPAA-compliant platform. Interviews were conducted by two study team members (EH, MS) who had no direct role in the Bio-Muse trial, minimizing the potential for bias. All interviews were audio-recorded, professionally transcribed, and de-identified for analysis purposes. As compensation for their time, each participant received a gift card following the interview.

### Data analyses

To facilitate the rapid identification of implementation strategies, we used a Rapid Qualitative Analysis approach [22]. Compared to more exploratory qualitative methods, rapid analysis is more explanatory, making it well-suited for efficiently describing an implementation environment and contextual factors that may influence that environment. To ensure rigor and guide our reporting, we used the Planning for and Assessing Rigor in Rapid Qualitative Analysis (PARRQA) framework and checklist [23].

Steps to the rapid analysis entailed:

1. A neutral domain name was created corresponding to each interview topic (and its associated CFIR construct), and a summary template for interview data was developed for team use.
2. Four team members (KMS, EH, KS, SLR) summarized the same interview to pilot the summary template and assess its usability and relevance.
3. Once consistency was established across the team, transcripts were divided among the same four team members and summarized.
4. One team member (KMS) created a matrix of all completed summaries using Microsoft Word. The matrix domains (9 total) were transferred from the CFIR-related domains used in the summary documents. Content from the transcript summaries was placed under the relevant domain.
5. The team met, discussed and iteratively refined the matrix (e.g., two domains were combined due to significant overlap).
6. A summary of the results was created and sent to research team members for confirmation.

## Results

Interviews were completed by ten individuals (91% of target sample) with representation from all three participating. One APN did not respond to the initial email invitation. The final sample of participants were all female, highly experienced in their field (> 6 years), and included two APNs (20%) and eight board-certified MTs (80%). Table 1 provides a detailed demographic summary of participant characteristics. The following narrative summary highlights common findings across all study sites and includes a representative quote for each construct. Following the narrative, Table 2 maps the findings to the CFIR constructs.

**Table 1:** Participant characteristics

Interviewee Characteristic	
**Hospital Position**	n (%)
Advanced Practice Nurse	2 (20%)
Creative Arts Therapy Program Manager	2 (20%)
Board-Certified Music Therapist	6 (60%)
**Study Role**	
Participant Recruitment/Coordination	2 (20%)
Music Interventionist	8 (80%)
**Years in Profession**	Median, (range)
Advanced Practice Nurse	6.5 years (6,7)
Music Therapist	12 years (6, 40)
**Degree**	
Doctor of Nursing Practice (DNP)	2 (20%)
Master’s Degree (MBA; MMT)	6 (60%)
Bachelor’s Degree (BMT)	2 (20%)

### Domain: Inner setting

#### Structural characteristics

The structural characteristics of large, fast-paced outpatient clinic settings presented specific implementation difficulties. These challenges centered primarily on securing dedicated physical space and scheduling dedicated time for sessions.

We have a fairly large clinic with multiple different oncology populations, and so being able to have just the physical space to be able to run this, it would take up a room in clinic during that time, and so that was a little bit more difficult to be able to say, “Hey, we need this room for an hour or so of time,” when other people are trying to see patients and get them kind of to and from there, that was a little bit more difficult in our clinic space. (ID 05)

Compared to an inpatient setting, the examination rooms where Bio-Muse sessions took place were noted as being much smaller, closer together, and sometimes simply bays closed off with a curtain. This lack of privacy meant sound traveled to other rooms, making others aware of the activity occurring in that space. Furthermore, the overall sense of urgency in the outpatient setting did not always align with the therapeutic intent and deliberate pacing of the intervention.

To facilitate implementation, participants suggested several potential solutions: establishing a dedicated MT position, educating outpatient staff about the MT role, and forming stronger relationships with care providers to better coordinate scheduling and space. They acknowledged that these changes would ultimately require the support of executive leadership at each institution (Table 2).

#### Relationships and communication

Participants highlighted the importance of relationships and clear communication in supporting the study, especially the ongoing need to educate staff and leadership about the music therapy role.

I feel most supported and connected to the people that really understand our role as music therapists. And a lot of our time is spent educating where there might be gaps in understanding of what our role is. And so, my team, who does what I do, I feel very supported and connected to. We’ve spent a lot of time with our current manager, who now, we feel like has a solid understanding of what we do and has become a really great advocate in that way. The farther up it goes, and I feel like there’s been less interaction between us directly. And so we feel like there might be some things that are lost in translation and more a generalization of what we do instead of a -- -- descriptive or a specific understanding. (ID 08)

Participants emphasized the strong relationships and clear communication that were essential for implementation. Most described communication as effective within their site teams, with frequent interactions among staff who worked closely with the music therapists. However, engagement with supervisory and executive leaders was more limited. At several sites, outpatient staff were initially unfamiliar with the MTs, but these relationships developed over the course of the study. While in-person communication was viewed as most helpful for building trust and collaboration, some teams relied on email and text messaging. Limited direct interaction was generally not perceived as a barrier because the roles were distinct and rarely overlapped.

While communication methods varied across sites and participants reported no major challenges during the trial, they did identify potential barriers for future implementation. Because MTs and APN had distinct roles in the Bio-Muse trial, they had limited direct interaction. Participants noted that building stronger relationships between MTs and general outpatient clinic staff could improve the visibility and integration of the MT’s role. Similarly, better rapport between APNs and MTs would likely have increased the MTs’ comfort in reaching out to APNs when issues arose, as some hesitated out of concern for the APNs’ demanding schedules.

For sustained implementation, participants recommended developing a collaborative learning community that includes MTs, APNs, physicians, nurses, support staff, and leadership. This community would facilitate knowledge exchange and shared problem-solving through regular online meetings, structured check-ins, and expert presentations. Participants emphasized the importance of addressing existing organizational hierarchies within this community to foster collaboration and respect among all members (Table 2).

#### Relative priority and compatibility

While the Bio-Muse trial took place in outpatient settings, participants frequently mentioned high compatibility of AME principles with the specific needs of inpatient children and parents. Participants also noted that AME’s emphasis on both child and parent was unique and important.

You have patients who come in, and typically this is from the three to five age range. And they come in and—the room is dark. They cry anytime someone opens the door, very hesitant around staff. No, no, no, no, no, anytime. They have a music therapy session with the AME, with the principles of getting to be in charge, getting to be the leader of music session, getting to be in control of their environment. And the next week, I see them roaming the halls and feeling like they’re more in charge and, like, they’re the boss and they’re running the show. In inpatient, that is so important because that is their new space… Outpatient, they come back and forth. It’s not like their home base. So outpatient to me feels different. I think there’s still a lot of value there, there’s really good outcomes whenever you’re adding music therapy into a space… I think inpatient, I just see that AME is like the lock and the key there for this age range. (ID 01)

While the Bio-Muse trial was conducted on outpatient units, participants recognized the broad applicability of AME principles across different patient populations and often referenced examples unique to inpatient settings when discussing its future implementation. MT participants noted that AME began to influence their practice outside of the research sessions:

I think probably without my even knowing it, AME kind of started to seep into my practice once I learned it and was able to implement it well within the context of those research sessions. It was even in my thinking outside of those sessions as well, and I think one of the reasons, in my opinion, it’s beneficial across the board and not just necessary for a certain target population is because its focus is so much on intervention design and crafting an intervention that meets targeted needs. (ID 11)

MTs noted AME’s strong underlying theory, embedded structure, and emphasis on family involvement as unique, beneficial components that continue to inform their work. The intervention’s structure and activities are guided by the Contextual Support Model of Music Therapy [8], which was particularly helpful for delivery. Parent involvement was repeatedly identified as a key concept, with many families engaging with the materials and activities outside of sessions. This combination of theoretical grounding and intervention structure was helpful to both new therapists, who could rely on the inherent structure, and experienced therapists, who could integrate the theory. Ultimately, AME provided growth opportunities for MTs across the practice continuum (Table 2).

#### Access to knowledge and information

Participants provided clear recommendations for the future of AME, emphasizing a collaborative training structure and the importance of creating adaptable intervention resources for wider use.

I think it was helpful to have other team members who were also doing the training at the same time to be able to practice things with them in person… I just wonder if this was going to be more widely implemented if the quantity of options for the different categories within AME would stay the same or if there would be in the future a resource library of like, here are all the different ways to refresh, you know, keep the structure the same but like different books, different like animal songs or whatever. (ID 07)

When asked how to best structure future AME training, participants emphasized the importance of receiving training as a community (as a group, alongside colleagues). They noted that focusing on the core concepts was essential for its successful application to other pediatric populations. Role-playing and video demonstrations were cited as especially helpful methods for understanding the AME concepts, and participants recommended creating a resource library to support future implementation.

Participants also shared suggestions to strengthen the training and support broader implementation. They recommended moving away from the manualized content and pre-prepared AME kit (used in the controlled trial) in favor of using more varied book and song choices that could be adapted for different ages, cultures, and diagnoses. They specifically noted that any adaptations should retain the theoretical constructs that underpin the AME intervention.

This emphasis on the importance of community and collaboration extended to their experience of knowledge sharing. Participants found the monthly team calls for study therapists to be highly valuable for peer support and problem solving:

When we were able to get on our monthly calls and discuss recent participants we’d had, we would often kind of veer into discussions about AME implementation just because that is mostly what we were working on and what we had questions about. So that opportunity to, again, like, consistently dialogue and also brainstorm with the team was very valuable because then you could get more insight into how other therapists start approaching it, maybe what’s been working for them, what needs to be retooled. (ID 11)

### Domain: Individuals, roles, and characteristics

#### Implementation leads

Participants reported a supportive and empowering environment, noting that some of their best advocates and champions were staff with whom they collaborated regularly. One participant explained:

I kind of came into this environment that was remarkably supportive and empowering of music therapy. I mean, some of our best advocates and champions are not even within our department. There are other staff that we work with and that we’re able to collaborate with pretty regularly in our everyday work. So I will say that overall our hospital, I would say personally, has a very wonderful view of music therapy and they support it, they ask for it, they champion it as an essential and valuable service. (ID 11)

MTs consistently reported that their strongest support came from colleagues in direct patient care roles. Clinic staff who were initially unfamiliar with music therapy or the Bio-Muse study became increasingly supportive over time as they learned more about the MTs’ role (Table 2).

#### Executive leadership

Participants perceived that executive leadership was less aware of music therapy services and research compared to lower-management and staff. There was a shared concern that leaders with primarily business backgrounds might foster a culture that created more inherent barriers to this type of research and service offering. One participant noted the contrast:

I feel really supported by, like, our team, like manager level down. And I think that our executive leadership is more focused on balancing the budget. They are building new hospitals and, you know, have a lot of more financial concerns and less concerned with patient outcomes at this time. (ID 01)

Participants believed that some of the leadership support, which included protected time for MTs to facilitate sessions outside of their usual areas of service, was largely due to the trial having external funding. Given that leadership is a key driver of successful implementation, some participants recommended a data-based approach to advocacy to ensure executive leaders are educated about MT services and research. This data would justify the growth of MT services by estimating demand and necessary staffing:

Is there a way that we could track some data at our site? How many kids on a weekly or monthly basis would fall into this category of really benefiting from AME? And then what does that translate to in terms of like the number of therapists we would need to actually carry that out?… I think we’ve had some success in that data-based approach to advocacy, (ID 07)

### Domain: Implementation process

#### Doing and adapting

Music therapist participants found the AME intervention easy to adapt while maintaining the integrity of the protocol:

I think most times, we were adapting in some capacity, but it was more just because I just happened to get to meet and work with a bunch of, very creative, funny children for lack of a better word…And so being able to adapt that in the moment, I think that was something that I relied on pretty heavily within my sessions just because it was naturally where the child was going. And within the bounds of the study, for the most part, you wanted to be able to honor that and give them that level of control. (ID 11)

MTs adapted the intervention for children at both the lower and upper ends of the trial’s target age range (3–8 years). Some suggested that expanding the AME kit to include more options would be beneficial, while others advocated for moving away from the pre-specified activities and kit altogether to work directly from the intervention’s theoretical constructs. Participants noted that MTs who were initially hesitant to make adaptations found reassurance and were able to generate new ideas through the trial’s monthly team calls. Newer music therapists, in particular, benefited from validation that enabled them to trust their clinical judgment without compromising the protocol:

I appreciated from my own rule follower way of being the opportunity to go back to the study team and ask questions about limits to which it would still qualify under the study. And really, my experience time and time again was that the Principal Investigator was always like, do what is best for the child, like stretch it really as much as it needs to be stretched, which I appreciated and I think is something that I grew into also as I grew as a clinician. So, I think that I benefited from reassurance that I could trust my clinical gut and that I wasn’t jeopardizing the integrity of what AME was. I think really like, just permission to slow things down or really simplify things. (ID 07)

MTs and APNs suggested that for future adoption, the intervention should be adapted for different cultures and diagnoses. Within outpatient settings, they also recommended modifying AME for use during procedural support, such as port access. They perceived that educating hospital staff and leadership about the benefits of AME, using data from the trial, will be important to expanding its application and adoption (Table 2).

#### Sustainability

Across interviews, participants identified several factors influencing the sustainability of AME implementation, primarily related to resources (staffing and supplies) and referral prioritization.

Resources. Participants emphasized that demand for AME far exceeds current staffing capacity. Although expansion was viewed positively, existing MTs are already at full capacity, necessitating additional positions and dedicated space. One participant summarized this concern: “I worry that the number of kids that could benefit…is far outside the realistic number that our staff could get to…it can be easier for us to get funding for stuff than for staff “(ID 07).

Participants also noted the need for ongoing funding for AME kit supplies. While sites often receive donations that could offset material costs, both staffing and supplies were seen as essential for sustainable delivery. Leadership advocacy at multiple levels was viewed as necessary for successful implementation.

Referrals and Patient Prioritization. Program managers stressed that while MT services already use waitlists and limit caseloads, successful broad implementation of AME would likely lead to an unmanageable influx of requests, necessitating clear prioritization methods. “We have a wait list that we use quite regularly… Once the word gets out that [AME] is an option… we’ll be inundated with requests… we just have to agree on how to handle [them]” (ID 02).

Referrals currently come from multiple channels—nurses, physicians, social workers, child life, families, and electronic health records. Some units have moved toward streamlined referral processes that rely on clinician discretion to determine the most appropriate service: “Stem cell would just automatically refer to all the creative arts therapies… We kind of stopped that… now we just get one referral… and determine which service best fits” (ID 02).

To manage the anticipated demand and improve patient referrals, participants recommended targeted education for referring staff to clarify which patients would most benefit from AME and the development of triage tools or assessment systems to guide referrals based on criteria such as age, treatment frequency, and diagnosis: “I would almost see [AME] as having its own triage tool…to guide what patients could benefit the most” (ID 07).

This type of structured decision tree was considered critical for successful long-term implementation, similar to how physicians use clinical paths for diagnosis and treatment.

I think of it like our physicians who use the clinical paths and the decision trees for their diagnosing and treatment. I think of that very similarly for us when we’re thinking of using AME more broadly as that standard. (ID 12)

**Table 2:** Themes mapped to CFIR domains

Construct	Rationale	Findings
**Domain: Inner setting.** AME was implemented in hematology/oncology outpatient hospital clinics.		
Structural characteristics	Infrastructure components support functional performance of the Inner Setting.	• Outpatient clinic space is busy with competing demands for the use of rooms. • Less space than inpatient rooms. • Closer proximity to other patients creates potential sound issues.
Relational connections	Formal and informal relationships support implementation and delivery of AME.	• Overall, relationships and communication were very good, especially with colleagues who overlap. If individuals were further removed from the MT, such as higher up supervisors or executive leadership, there was less interaction. • In-person contact facilitates relationship building. • MTs and APNs were not in relationship with each other at some sites.
Communications	Formal and informal sharing practices supports implementation and delivery of AME.	Please tell me about any meetings, such as staff meetings, held regularly (prompts: who attends, are they helpful, how are they structured). When you need to get something done or to solve a problem, who are your “go-to” people? (It may be helpful to ask for a recent example.)
Relative priority	To establish the importance of implementing AME compared to other similar interventions (uniqueness).	• AME underlying theory of contextual support. • AME structure is helpful. • AME’s involvement of caregivers (family) in the session was frequently mentioned. • Families’ engagement with materials/practices outside of sessions. • The way in which training in AME and the constructs inform therapists’ other sessions.
Compatibility	To determine how well AME fits within the hospital and unit’s workflows and processes.	• AME could benefit patients in outpatient and inpatient • Music Therapists aren’t currently working in outpatient clinics at many hospitals and likely don’t have staffing available to take on more patients. • Space and time tend to be tight in outpatient clinics, making AME more challenging, especially outside of the study. Noise can also be an issue in small clinic spaces. • AME fits well into current inpatient music therapy practice and workflow.
Access to knowledge & information	To determine accessibility and relevance of AME training and guidance.	• Training in community is important. • Core concept training is important because the concepts can then be applied to different populations. • Role playing and videos are helpful • Suggestions for an ongoing resource library.
**Domain: Individuals**,** roles**,** and characteristics.** Interviewees were implementation leads, supports, and music therapists. We inquired about relationships and support with mid-level and high-level leaders.		
Motivation of executive leadership	Mid-level and high-level leaders have authority to make decisions that may affect implementation of AME.	• Leadership was less aware of AME. • Some concern that executive leaders with business backgrounds would promote a culture with more inherent barriers. • More support and champions from those in direct care roles.
Implementation leads	Perception of available champions to promote implementation of AME	
Capability of implementation and delivery	Self-perception of ability to implement and sustain AME	• Resources for more MTs (dedicated positions?) to expand services. • Hospitals would need resources to fund AME kits to continue to provide to families. • Supplies are easier to support than positions.
**Domain: Implementation process.** Activities and strategies used to implement AME		
Doing and adapting	Determine to what degree AME was adapted or modified for optimal delivery	• Some MTs said more book/song choices would be helpful. while • Others suggested moving away from the kit and working from theory constructs. • Adapt for different ages, cultures, and diagnosis. • Adapt a shorter intervention for support during port placement
Sustainability	Technically an implementation outcome but helps determine potential barriers and facilitators sustaining implementation.	• MT services are mainly at capacity, with a waiting list. • Strategies to facilitate implementation include triaging referrals to those patients who would benefit the most or hiring more music therapists to meet the need.

## Discussion

In this study, we used CFIR to identify barriers and facilitators for implementing the Active Music Engagement protocol in three pediatric hematology/oncology healthcare settings. Our findings provided important insights into the current barriers and facilitators of AME implementation, mapped to the three CFIR domains of Inner Setting, Individual Roles and Relationships, and the Implementation Process. Researchers have previously established that successful implementation of evidence-based interventions is dependent on factors like effective leadership, staff and leadership engagement, and a supportive organizational climate [24, 25]. Our findings partially align with these determinants, as we found strong support for AME among those who had direct exposure to it. However, therapists expressed concern that hospital leadership – particularly those responsible for resource allocation and decision-making – lacked awareness of the study protocol and evidence about its benefits for parents and children. We discuss three primary findings from this analysis: (1) where to best locate AME delivery (inpatient vs. outpatient) for optimal adoption and reach (2), the need for enhanced referral systems (3), essential features for therapist training and strategies for building a strong stakeholder community.

AME has been studied in both inpatient and outpatient care settings [11, 12, 16, 26]. As we prepare for a large-scale implementation trial involving a broader population of patients and families, it is important to determine where AME best aligns with current patient care workflow, resource availability, and existing music therapy service structure. Our analysis, focusing on the Inner Settings domain, revealed significant barriers to sustained delivery in the outpatient care setting, including competing demands on patient time and limited clinical space. Additionally, most U.S. pediatric music therapy programs do not offer outpatient services [27, 28]. As a federally funded multi-site trial, we had resources to support therapist time and effort in the outpatient clinic and secured support from physicians, nurse managers, care coordinators, and clinic staff to help us integrate AME delivery. However, our interviews revealed that the sustainability of AME in this setting would require a dedicated music therapist position, time and space for service delivery, and ongoing efforts to establish music therapy as a fully integrated service. Together, these findings suggest that AME is best suited for delivery in inpatient care settings where music therapy is already an established service and is likely to have the greatest reach and impact, especially for the families that research suggests are most likely to benefit [27–29].

Ensuring that AME reaches the families who can benefit most requires development and implementation of enhanced referral systems. Findings from our pre-implementation evaluation highlighted two critical areas for development: enhanced educational initiatives to promote appropriate referrals and the use of standardized assessment tools to improve existing service prioritization. Interview participants stressed that without clear prioritization tools and capacity planning, an increase in referrals could risk staff burnout and compromise quality of care. They also noted that better prioritization strategies could aid program growth by providing essential data on the proportion of families meeting referral thresholds for AME and the associated costs, thereby supporting program expansion.

Our analysis, combined with findings from a national cross-sectional survey of pediatric music therapy programs, underscores the urgent need for focused work on developing enhanced referral and prioritization systems [28]. Music therapy services are rapidly becoming a standard of care in U.S. pediatric hospitals, with hematology/oncology frequently identified as a primary unit for service delivery. Despite this growth, the ratio of music therapists to patient beds remains high (1:86), with therapists often covering multiple units. While most referrals (63%) originate in the electronic medical record, the remaining are verbal. Subsequent prioritization occurs at the program or therapist level, but the procedural processes used are not well known or described in published works. A recent publication by Bower and colleagues describes their hospital’s prioritization framework and discusses how these systems are essential for effective resource allocation, maximizing patient benefit, and mitigating the moral distress clinicians can experience without this [30]. As evidence emerges to predict who benefits most from a specific music intervention, the integration of screening tools will further refine these prioritization systems.

In our randomized controlled trials, we trained therapists on the AME protocol using a standardized set of music experiences and a strong emphasis on core theoretical principles [11]. To ensure fidelity and rigor, therapists also participated in ongoing fidelity monitoring and monthly calls [20]. While these methods are essential for maintaining research integrity in a clinical trial, they are often difficult to generalize to broader clinical practice [31, 32].

Our analysis, focusing on the *Implementation Process* domain, helped us identify strategies for successful adoption. Consistent with implementation science, our therapists emphasized the need for training that focuses on the practical application of core theoretical principles [33, 34].This approach would support therapists to move beyond the specific music experiences used in the randomized controlled trial and tailor the intervention to a broader population of children and families. To better facilitate internalization and application of this knowledge, therapists recommended that future training incorporate theory-based tools, case examples, role-playing, and video demonstrations. They also suggested developing an online resource to support tailored delivery based on a child’s age, developmental level, music preferences, and cultural background. Finally, to prevent professional isolation and foster competence, they recommended creating a community of learners through individual consultations and learning collaboratives.

### Limitations

The scope of this rapid analysis was limited to those directly involved in AME delivery during the Bio-Muse trial. Mid- and high-level leaders with decision-making authority regarding music therapy referral processes, patient prioritization, and resource allocation were not included in this phase of the study. Future evaluations should engage a larger sample of hospitals and incorporate broader representation from these key stakeholder groups to advance understanding of leadership culture and capacity for resource expansion. Furthermore, because AME is a dyadic music intervention that benefits parents, a deeper understanding of how current music therapy services address the needs of parents and caregivers and whether they are included in the prioritization process is warranted.

## Conclusions

This study offers an important contribution to the literature by highlighting the value of pre-implementation research and providing an exemplar for other pediatric behavioral health investigations. Guided by the CFIR framework, our rapid analysis identified key contextual barriers and facilitators to broader implementation of AME within pediatric hematology/oncology settings. Findings indicate that to promote sustainability, AME delivery should be integrated within existing inpatient infrastructures, supported by enhanced referral and prioritization systems, and strengthened through refined therapist training and staff engagement. Future research should engage leadership and parents to develop scalable strategies for integrating AME into routine inpatient pediatric care.

## Supplementary Information

Below is the link to the electronic supplementary material.

Supplementary Material 1

## Data Availability

The datasets used and/or analyzed during the current study are available from the corresponding author on reasonable request.

## Abbreviations

AME: Active Music Engagement
APNs: Advanced Practice Nurses
Bio-Muse: Biologic Mechanisms and Dosing of Active Music Engagement
CFIR: Consolidated Framework for Implementation Research
HIPAA: Health Insurance Portability and Accountability Act (HIPAA-compliant platform)
ID: Identifier
MT: Music Therapist
PARRQA: Planning for and Assessing Rigor in Rapid Qualitative Analysis (framework and checklist)
TSS: Traumatic Stress Symptoms
