# Supplementary material for: Pre-implementation factors for Active Music Engagement (AME): barriers and facilitators to integrating an evidence-based dyadic intervention into pediatric oncology care
Source: Implement Sci Commun. 2026 Jun 3;7:146. doi: 10.1186/s43058-026-00979-y (PMC13466145; doi:10.1186/s43058-026-00979-y)
Supplement: Supplementary file 1 [file 43058_2026_979_MOESM1_ESM.docx]

Supplementary file 1. Interview Guide

**AME Interview Guide**

*“Thank you for taking the time to speak with me today. Our research team is interested in your feedback about the Active Music Experience (AME) intervention that was delivered at your facility in the context of the Bio-Muse research study. We would like to record this interview for transcription purposes. We will remove any identifiers from the transcript and* *delete the recording once it has been transcribed. Do I have your permission to record this interview for research purposes?”* Start RECORDING

*Today is (date) and this is participant #( …).*

*We are interested in receiving your feedback based on your unique role at your facility and your involvement with the AME intervention. I’d like to start with a few context questions. (FOR MTs: Generally asking about your specific role with AME, as opposed to music therapy as a whole, although that may provide some insight into the culture of the facility/ team)*

1. What is your role at the hospital?

2. How were you involved with AME?

Great, is there anything you’d like to tell me about AME and your experience with it, before we move onto some specific questions?

I’d like to move on to questions that may help us identify some barriers and facilitators to future implementation of AME.

**Structural Characteristics**

1. In what ways, if any, do you think the infrastructure of your hospital (age of the building, size, or physical layout) affected the implementation and delivery of AME?
   a. If challenges are identified, ask about workarounds/ solutions.

**Relational Connections**

1. Can you describe your working relationships with your colleagues (on your unit and more widely at the hospital)?
   a. It may be informative to ask about a “time you needed to work with others to solve a problem? Or resolve a challenge related to BIO-MUSE/PINPOINT?”
2. Can you describe your working relationship with leaders (direct supervisors as well as more executive leadership?)

**Communications**

1. Please tell me about any meetings, such as staff meetings, held regularly (prompts: who attends, are they helpful, how are they structured).
2. When you need to get something done or to solve a problem, who are your "go-to" people? (It may be helpful to ask for a recent example.)

**Relative Priority**

1. What is the general level of receptivity in your hospital to music therapy in general and more specifically to implementing AME?

**Tension for Change**

1. Is there a strong need for AME at your hospital?
   a. Why or why not?
   b. Do you think others at your hospital see a need for it?

**Compatibility**

1. How well does AME fit with existing work processes and practices in your setting?
   a. What are likely issues or complications that may arise?
2. Will the intervention replace or compliment a current program or process?
   a. In what ways?
3. If you were involved with AME in more than one setting (inpatient/outpatient), what were some of the differences?
   a. Did one seem to be a better fit, if so, why?

**Access to Knowledge & Information**

1. What kind of training did you receive? (for non-therapists, ask about education and information about AME)
   a. Do you feel the training will prepare you to carry out the roles and responsibilities expected of you? Can you explain?
   b. What are the positive aspects of planned training?
   c. What is missing?
   d. Do you think there is a need for continued training is planned?
   e. Something here about essential components of the training (as related to essential components of the intervention)

**Leadership Engagement**

1. What level of endorsement or support have you seen or heard from leaders?
   a. Who are these leaders and how has this affected things so far? Going forward?
   - Are there barriers/ challenges you expect to encounter moving forward?

**Capability (Self-efficacy)**

1. How confident are you that you will be able to successfully implement/sustain AME?
   a. What gives you that level of confidence (or lack of confidence)?

**Implementation Leads (Champions)**

1. Who are the people in your organization who are likely to champion (go above and beyond what might be expected) promoting AME?

**Doing and adapting**

1. Has AME been implemented according to the research plan? Were there modifications or adaptations you needed to make (within the context of the protocol) to accommodate patients, families, or other staff and care needs?
2. Are there modifications or adaptations that you think would make AME easier to use outside of the research context?
